# WHO Surgical Checklist and Its Practical Application in Plastic Surgery

**DOI:** 10.1155/2011/579579

**Published:** 2011-04-27

**Authors:** Shady Abdel-Rehim, Andrew Morritt, Graeme Perks

**Affiliations:** Department of Plastic Surgery, Nottingham University Hospitals NHS Trust, City Hospital Campus, Hucknall Road, Nottingham NG5 1PB, UK

## Abstract

The WHO surgical checklist was introduced to most UK surgical units following the WHO “Safe Surgery Saves Lives” initiative. The aim of this audit was to review patient's safety in the delivery of surgical care and to evaluate the practical application of the new WHO surgical checklist. We conducted a retrospective audit of patients who received operative treatment under general anaesthesia at our Plastic Surgery Department, involving a total number of 90 patients. The WHO form was compared to its former equivalents. Complications or incidents occurring during or after surgery were recorded. Using the department's previous surgical checklist, “Time out” was only performed in only 30% of cases. One patient arrived at theatre reception without a completed consent form, and two clinical incidents were reported without patients suffering harm. Following introduction of current WHO surgical checklist, “Time out” was recorded in 80% of cases. In all cases, the new WHO surgical checklist was used and no incidents were reported. The WHO surgical checklist provides a structured frame work that standardizes the delivery of care across hospitals and specialized units; however, it will take some time and practice for teams to learn to use the checklist effectively and reliably.

## 1. Introduction

**Figure 1:**
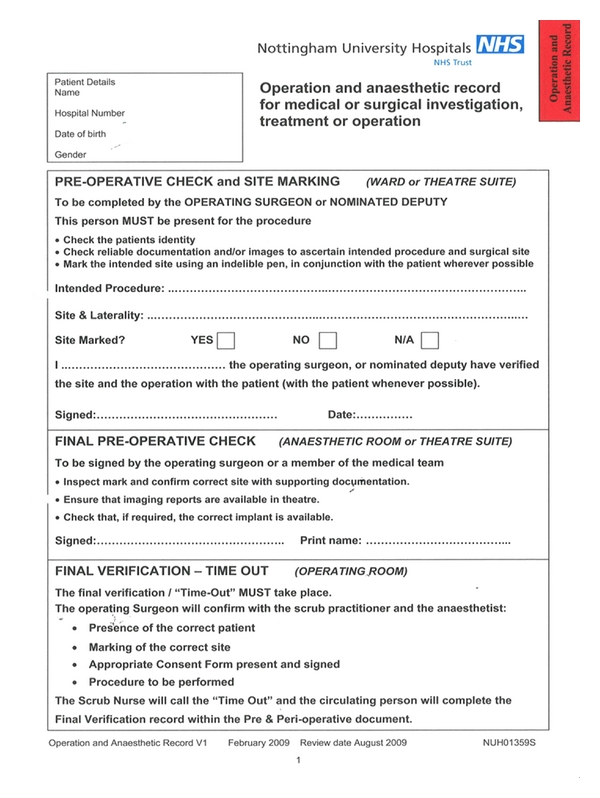
Nottingham University Hospitals Trust surgical checklist.

Patient safety forms an integral part of any healthcare organization. Unfortunately, surgery can be an unsafe environment and provide a unique opportunity for adverse effects. In the UK, over one million incident reports have been collected since the National Patient Safety Agency was founded, and between 1 April 2007 and 31 March 2008, there were 135,247 incidents reported in surgical specialties to the Reporting and Learning System (RLS) [1]. In June 2008, the WHO launched a second Global Patient Safety Challenge, “Safe Surgery Saves Lives,” to reduce the number of surgical deaths across the world. The initiative aimed to identify minimum standards of surgical care that could be universally applied across countries and settings. One component of the initiative was the introduction of a perioperative checklist [2]. The National Patient Safety Agency (NPSA) in the UK has issued an alert requiring all hospitals in England and Wales to implement the peri-operative checklist by February 2010 [1]. Prior to the introduction of the WHO surgical checklist, the peri-operative checklists developed according to the local trust policy were used. The local annual audit review at our hospital identified areas where standards were not met and other areas of shortfalls. The peri-operative checklist mainly consisted of three sections: “Pre-operative/ward check”, “Anaesthetic room check”, and “Final verification/Time out”, with its main focus on completion of patient records, marking, and documentations; however, it failed to address other clinical areas for example, anticipation of airway problems, blood loss, thromboembolism, and administration antibiotic prophylaxis (Figure 1).

**Figure 2:**
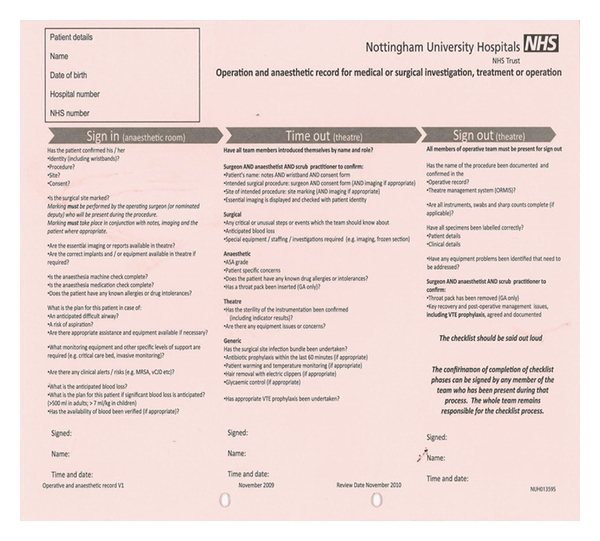
WHO surgical checklist, Nottingham University Hospitals version.

The WHO surgical checklist is globally applicable to most surgical procedures. It involves a 19-item checklist and allows consistency of care reducing complication and death rates [3]. It is used at 3 junctures:-before the induction of anaesthesia “sign in”, before incision of the skin “time out”, and before the patient leaves the operating room “sign out”. Each step is “read out” allowing active communication among operating team and rest of theatre staff (Figure 2). 

The aim of our audit was to review patient safety in the delivery of surgical care and to assess the practical application of the new WHO surgical checklist. Following our audit results, we have participated in a national piloting scheme of WHO surgical checklist prior to its official use. This was followed by further auditing of our practice using the WHO surgical checklist as it became officially a national policy.

## 2. Methods and Results

Over a period of five months, from September 2009 till January 2010, we conducted a retrospective audit before and after implementation of the WHO surgical checklist. We examined the hospital records of patients undergoing surgery under general anaesthesia. Ninety consecutive patients were included, 48 males and 42 females, age range between 8 to 96 years with average age of 43.1 years. The American Society of Anesthesiologists (ASA) grading for anaesthetic assessment ranged between I–III. Due to the different nature of surgical procedures performed, we have subdivided all operations into three main categories: elective, emergency, and day case procedures, Table 1.

In addition to the retrospective case-note analysis, minutes and documentations from our monthly mortality and morbidity meetings were examined. Records of the operating theatres as well as any incident forms involving clinical incident or a “near miss” reported to the clinical director of our plastic surgery department were also reviewed.

**Figure 3:**
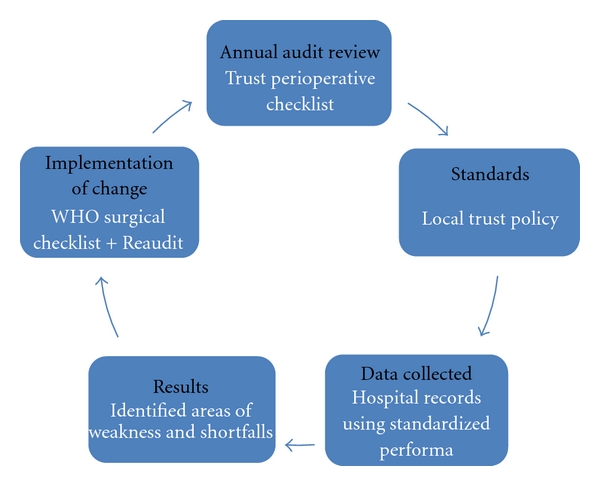
Audit cycle module.

Data were collected on standardized Performa and Excel spreadsheet. The percentage of correct entries was calculated for each item. We have followed the five-step module of an audit cycle (Figure 3).

During the whole process, before and after implementation of changes (WHO checklist), we looked at the following: “Sign in” (prior to induction of anaesthesia), “Time out” (prior to skin incision), and “Sign out” (after surgery before the patient leaves the operating theatre), on the new form and its equivalents on the local department's checklist, as well as any complications or incidents occurring during or after surgery. 

**Figure 4:**
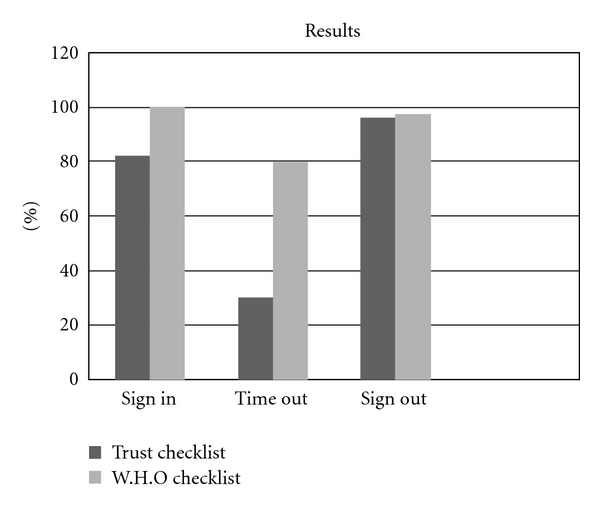
Before and after implementation of WHO surgical checklist September 2009–January 2010.

Although the two checklists are not identical in their contents, one can generally categorize their contents into the following three sections: “Sign in” (prior to induction of anaesthesia), “Time out” (prior to skin incision), and “Sign out” (after surgery before the patient leaves the operating theatre), as illustrated in the graph (Figure 4).

The WHO surgical checklist unlike the standard local trust form provides a tool for risk assessment of anaesthetic machine, patient allergy, airways problems, anticipation of amount of blood loss, antibiotics prophylaxis, and thromboprophylaxis as well as any extra or unusual surgical steps. Using the department's previous surgical checklist, 82% of patients had their documentation checked by the surgeon or *nominated deputy* on the ward. Only 30% of patients had their documentation rechecked in the anaesthetic room. “Sign out” was completed successfully for 96% of patients. One patient arrived to theatre reception without a completed consent form, and two clinical incidents were reported without patients suffering harm. After implementation of the current WHO surgical checklist, all patient consent forms, marking, and documentation were checked prior to surgery by *operating surgeon*. In 80% of operations, “Time out” was recorded, and in one case the operating surgeon did not participate in sign out. In all cases, the new WHO surgical checklist form was used and no incidents were reported.

## 3. Discussion

Donaldson et al. estimate that a degree of error occurs in 5–15% of all hospital admissions Worldwide [4]. It is estimated that 45% of medical errors occur in the operating theatre [5], with almost half of these being preventable [6]. The peri-operative checklist in health care systems is based on similar systems used in high risk industries such as aviation and nuclear power. The power of these checklists is to develop clear defined strategies and procedures in order to identify and avoid potential risks [4]. 

Following the introduction of the WHO peri-operative checklist, there is evidence that the rates of death and complications among patients over16 years of age and undergoing noncardiac surgery in a diverse group of hospitals has significantly decreased [3]. Our audit investigating the use and importance of the WHO surgical checklist in a specialized Plastic Surgical unit illustrates the significant positive outcome of the use of this procedure in theatre has brought about. The current increasing number of surgical procedures combined with the high volume of patient turn over in particular day case patients increases the risk of errors and potential morbidity or mortality. Moreover, shorter working hours, shift systems, staff limitations, multiple handover, and frequent change of staff may also contribute to this phenomenon. The diversity of race and nationality of theatre personnel may result in communication problems and this too may contribute to mistakes occurring in theatre. These features of modern health care organizations both nationally and internationally brought the need to implement robust systems of measurable and reproducible steps allowing effective and safer delivery of care. The application of these safe mechanisms in the surgical theatre allows best practice and significantly decreases the number of human errors. The corner stone to the success of the WHO checklist is team work and continuous communication. This guarantees improved surgical procedure and better outcomes. The surgical checklist gives the opportunity to get all personnel involved in the management of the patient, the operating surgeon, anaesthetist, scrub nurse, and operating theatres practitioners, to check and countercheck any actions or interventions before they are carried out. The checklist involves repetition, rehearsal, and vocalisation of these maneuvers by the members of the team improving the probability of good performance [7]. Similar actions were previously encouraged, especially the ‘‘Surgical pause” introduced by former UK Health Minister Lord Darzi in 2004 which provides a final check in the operating theatre, by anaesthetic, surgical, and nursing staff prior to the commencement of surgery [8, 9].

The need to introduce a new checklist stemmed from the inadequacies inherent to the local trust checklist. The initial process of signing in the patient was often carried out in the ward rather than in the operating theatre and often by a junior doctor rather than the operating surgeon or the team present in theatre. This inherently natured the risk for error and miscommunication. Moreover, recent observations reveal that the pre- and postsurgical checks of the patient were also often missed or carried out in the anaesthetic room, and on many occasions, the members of the team including the scrub nurses were thus not involved in this process. 

The WHO checklist is a generic risk assessment tool that can be used with most of interventional treatment and within all surgical specialties. Plastic Surgery involves wide range of surgical procedures from a small lump removed under local anaesthetic to a major reconstructive procedure requiring several hours in the operating theatre. Long hours in the operating theatres do not go without risks, and the technical part of the surgery may just represent one part of the whole process. Formation of a clot or an embolism may lead to serious complications and fatal outcomes. Even minor plastic surgery procedures pose its own risks; wrong site surgery is just one example of what can go wrong. With an extensive and endless list of risk factors, plastic surgery should not be considered to be any less of a risk than any other type of surgery, and full implementation of risk assessment tools including surgical checklists is recommended in order to minimize adverse effects. 

The N.P.S.A. (National Patient Safety Agency) has issued a manual which highlights the correct application and use of the WHO checklist. It emphasises that the protocols and guidelines devised for use in the surgical department would only generate the improved results through communication and team work if they are correctly applied. 

Our hospital clinical risk committee co-ordinates activity relating to risk and governance and oversees the operational delivery against agreed plans. Following the WHO recommendations and participation in the pilot phase, the WHO surgical checklist became a mandatory risk assessment tool required for all surgical procedures within the trust. As with the introduction of any new policy, all health care professionals related to operative field underwent induction training of how to use and effectively implement the changes required by the new surgical checklist. Fortunately, participating in the pilot phase has facilitated the introduction of those changes, which were widely accepted by most of the surgical staff. Further questionnaires and interviews among surgical staff might be required to gather more feedback on the use of the WHO surgical checklist.

Current surgical trainee curriculum focuses on the trainee's importance of developing not only clinical and surgical technical skills during their structured training but also nontechnical skills including situation awareness, teamwork, effective communication, decision-making, and leadership [6]. These skills are vital in the prevention of human error and hence unnecessary patient morbidity and mortality. Regular use of these checklists promotes team working environment and effective communication, thus allowing surgical trainees to practice and acquire those skills throughout their training.

## 4. Conclusion

Checklists provide a valuable tool in minimizing human error in modern surgical theatres. Their long term use in other high risk industries and within hospitals, particularly critical and intensive care units, and their significant benefit have prompted their use in general patient hospital intervention. The checklist provides a structured framework that standardizes and regulates the delivery of care across hospitals. Furthermore, they help to ease communication between staff members and encourage team work. It can aid to reducing hierarchy and the fear of speaking up, preparing team members for the expected as well as the unexpected [10]. To allow the WHO surgical checklist to be well understood and carefully applied, the operating teams should be given adequate training in developing these vital nontechnical skills, and more importantly the results of the intervention (either positive or negative) should be fed back to the teams to allow change in practice if required [10]. Our audit supports the use of the WHO surgical checklists and highlights its relevance and correct methods of application in surgical practice. We also stress on the importance that checklists should not be taken as tick box exercise which can result in counterproductive effects of no relevance and loss of its objectives. However, it will take some time and practice for teams to learn to use the checklists effectively and reliably.

## Figures and Tables

**Table 1:** Types of surgery performed.

Type of surgery	Number of patients (%)
Elective	47 (52.3)
Emergency	33 (36.6)
Day case	10 (11.1)

Total	90 (100)

## References

[1] National Patient Safety Agency (NPSA) The safer surgery alert. http://www.nrls.npsa.nhs.uk/.

[2] World Health Organisation (WHO) Safe surgery saves lives. The second global patient safety challenge. http://www.who.int/patientsafety/safesurgery/en/index.html.

[3] Haynes AB, Weiser TG, Berry WR (2009). A surgical safety checklist to reduce morbidity and mortality in a global population. *The New England Journal of Medicine*.

[4] Donaldson LJ (2007). The quest for safer surgery. *Surgeon*.

[5] Flin R, Yule S, McKenzie L, Paterson-Brown S, Maran N (2006). Attitudes to teamwork and safety in the operating theatre. *Surgeon*.

[6] Emerton M, Panesar SS, Forrest K (2009). Safer surgery: how a checklist can make orthopaedic surgery safer. *Orthopaedics and Trauma*.

[7] Clarke JR, Marella W, Johnston J, Davis M (2005). A surgeon who CARES can be safer. *American Journal of Surgery*.

[8] Bann S, Darzi A (2004). A protocol for the reduction of surgical errors. *Quality and Safety in Health Care*.

[9] O'Connor T, Papanikolaou V, Keogh I (2010). Safe surgery, the human factors approach. *Surgeon*.

[10] Vijayasekar C, Steele RJC (2009). The World Health Organization's surgical safety checklist. *Surgeon*.

